# Long-Term Outcomes Following Suppressive Antibiotic Therapy: A 10-Year Cohort Study

**DOI:** 10.3390/antibiotics14111164

**Published:** 2025-11-17

**Authors:** Ruth George, Christopher Kiss, Ian Woolley, Jillian S. Y. Lau

**Affiliations:** 1 Monash Infectious Diseases, Monash Medical Centre, Monash Health, Clayton, VIC 3168, Australia; ruth.george2@monashhealth.org (R.G.);; 2 Faculty of Medicine, Nursing and Health Sciences, Monash University, Clayton, VIC 3800, Australia; 3 Department of Infectious Disease, The Peter Doherty Institute for Infection and Immunity, The University of Melbourne, 792 Elizabeth St., Melbourne, VIC 3010, Australia

**Keywords:** antibiotics, lifelong, suppression, antimicrobial resistance, deprescribing, SAT, suppressive antibiotic therapy

## Abstract

**Background**: Lifelong antibiotic therapy can be used as a strategy to manage chronic infections deemed incurable. While this may be beneficial in suppressing infections, its long-term consequences remain underexplored. **Methods**: Conducted at a tertiary healthcare network in Melbourne, Australia, this 10-year longitudinal observational study, starting in 2015, followed up an initial cohort of 29 patients prescribed suppressive long-term antibiotics. Data extracted from medical records included patient demographics, antibiotic use, adverse events, mortality, and Charlson Comorbidity Index scores. Outcomes were assessed using descriptive statistics. **Results**: Of the original cohort of 29 patients in 2015, 19 were still alive at the five-year follow-up, with 14 of those continuing antibiotic therapy. At the 10-year follow-up, an additional three patients had died (total 11/29; 38%), and one had ceased antibiotics (total 6/29; 21%). Notably, none of the patients who had previously ceased antibiotics resumed therapy. Four patients were lost to follow-up, and only two patients were seen by infectious disease specialists for their long-term antibiotic therapy. Ultimately, of the original 29 patients initially recruited, only seven patients were confirmed to remain on antimicrobials by the 10-year follow-up. **Conclusions**: This 10-year prospective study highlights the complexities of lifelong antibiotic therapy. While some patients may benefit from prolonged antibiotic therapy with prevention of relapsed infection, the high burden of comorbidities, therapy adjustments, and hospitalizations remains a challenge. Long-term care strategies and individualized treatment approaches are essential. Further research is needed to optimize outcomes and refine criteria for lifelong antibiotic use and its management.

## 1. Introduction

Antibiotics were originally developed with a view of short-term curative therapy for bacterial infections [1,2]. With time, evidence grew for the use of prolonged antibiotic courses as an effective strategy in prophylaxis against infections [3]. The use of long-term antibiotic therapy is emerging as a recognized strategy for managing chronic infections that are deemed incurable [4]. They are often used to suppress infections involving prosthetic materials, such as vascular grafts or cardiovascular implantable electronic devices, when source control is infeasible [4]. Suppressive therapy is typically considered after an initial induction phase of antibiotics to control infection, with a choice based on microbial susceptibility, safety, and patient tolerance [5,6]. Studies suggest varying success rates in the use of suppressive therapy, and there remains limited evidence regarding the long-term outcomes and potential risks associated with prolonged antibiotic use. These studies have also described the association of long-term antibiotics with adverse effects—the most common of which are gastrointestinal issues, but also include skin rashes, cytopenia, and *Clostridioides difficile* infection [4,7,8,9,10]. Other concerns include a disruption of gut microbiota and promotion of antimicrobial resistance, which may make future infections harder to treat [10].

A previous audit of antibiotic prescribing practices at Monash Health, a large tertiary hospital network in metropolitan Melbourne, Australia, identified patients prescribed antibiotics for longer than 12 months to suppress chronic infection or prevent recurrent infection where definitive source control was unable to be achieved (e.g., infected prosthetic, recurrent bacteremia, and osteomyelitis). From this audited cohort, 29 patients were opportunistically enrolled into a longitudinal observational cohort study from the Infectious Diseases clinic at the main Monash Health Campus. At the five-year mark, two patients had moved to health services and were excluded from follow-up. Of the remaining 27 patients, 30% (8/27) had died, 18% (5/27) ceased antibiotic therapy, and only 52% (14/27) remained on long-term antibiotics. Eight patients developed MDRO colonization while receiving suppressive therapy; it was detected between initiation and the five-year follow-up. This was likely underestimated due to the absence of routine MDRO screening [11]. Overall, the study underscored the need for ongoing surveillance and evaluation to optimize therapeutic outcomes and minimize adverse effects.

Therefore, the aim of this study was to evaluate the 10-year outcomes of patients prescribed prolonged suppressive antibiotic therapy, specifically examining treatment continuation rates, treatment modifications, adverse events, and mortality. By examining the extended outcomes over a decade, this study offers critical insights into the risks and benefits of lifelong antibiotics, contributing to improved clinical decision-making in managing chronic infections.

## 2. Results

### 2.1. Patient Demographics

Patient outcomes are summarized in Table 1. Of the original 29 patients enrolled, two had no follow-up through our institution and were excluded from the analysis. At the five-year mark, eight patients had died, leaving only 19 for follow-up in the analysis. The median age of this surviving cohort was 68 (range 47–88 years old), and 10 were female (53%). Of those continuing antibiotics after five years, eight different antibiotic regimens were used, with a combination of rifampicin and Fusidic acid (36%) and cefalexin monotherapy (21%) prescribed most. Indications for long-term antibiotic prescriptions were diverse, the most frequent being prosthetic joint infection (PJI) (53%) and vascular graft infection (VGI) (16%). The median Charlson Comorbidity Index (CCI) changed over the study period from five (range 1–13) at baseline to seven (range 1–13) by the end of the five-year follow-up and six (range 2–13) by the end of the 10-year follow-up.

### 2.2. Patient Outcomes

**Figure 1:**
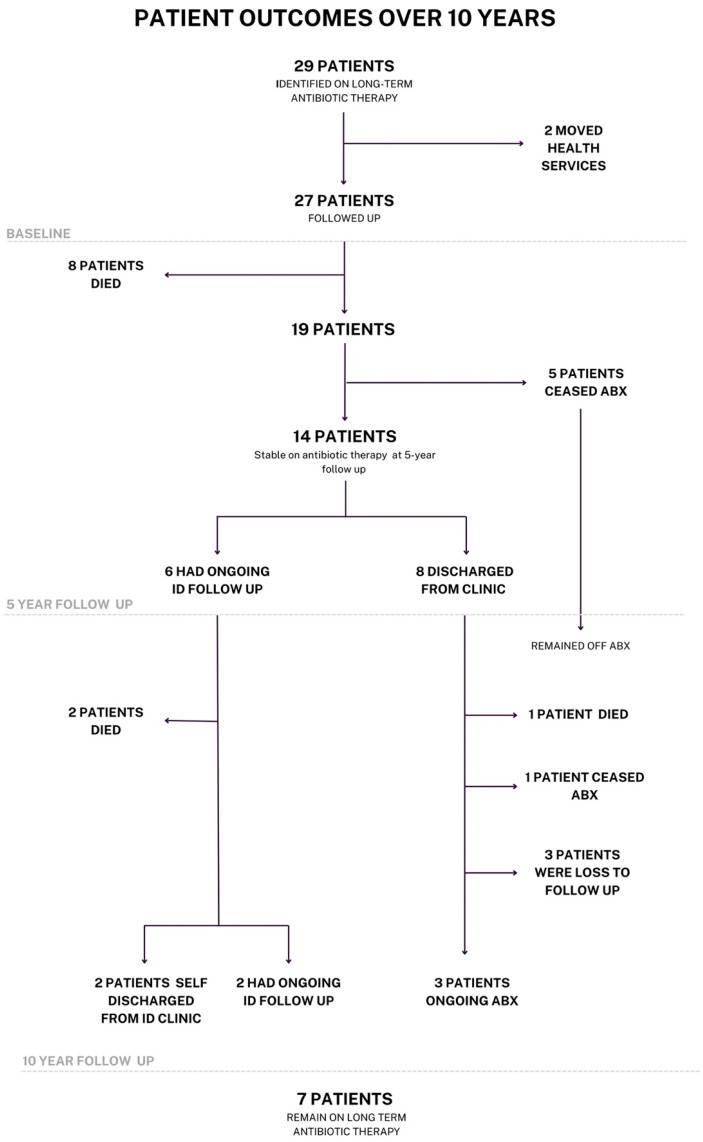
Patient outcomes over 10 years. ADR, adverse drug reaction; ID, infectious diseases.

Patient outcomes are summarized in Figure 1. At the 10-year follow-up, a further 3 patients died (total of 11/27—41%). One of whom died from infection (unrelated to their suppressed infection), the other of metastatic cancer, and another on whom no data was available. The first two patients still had ongoing ID follow-up and were stable on their antibiotic regimen at the time of their death. Only one further participant ceased their antibiotics for unknown reasons—they did not have ongoing infectious disease follow-up at the time of this change; however, they were seen regularly by different specialist teams within the health network (total of 6/27—22%). A further participant had a regimen adjustment due to medication shortages, and three patients were lost to follow-up. Two patients elected to self-discharge from the infectious diseases clinic—in both cases, this was due to other comorbidities. However, both patients continued their suppressive antibiotic therapy, which was managed by their general practitioner (GP). Of the original cohort, only seven patients remained on antibiotics, and only two had ongoing infectious disease follow-up, with annual reviews. The others remained stable on their antibiotics and were managed by their primary care physician. Of these seven, only four remained on their original antibiotic regimen. Notably, none of the patients who had ceased their antibiotics had to return to therapy for relapsed disease.

### 2.3. Adverse Events

Eleven patients required hospital admission between the five-year and 10-year follow-up period. Four of them had an infection unrelated to the one requiring suppressive therapy. No patients reported any further adverse drug reactions related to their antibiotic regimen. All the reported adverse drug events were noted during the original study. No patients re-presented to our hospital network with evidence of MDROs, and no routine screening was completed.

## 3. Discussion

Suppressive antibiotic therapy is largely prescribed in patients for whom definitive source control, often through surgery, cannot be achieved. As an extension to the five-year follow-up of outcomes in patients on lifelong antibiotics study, the 10-year follow-up study demonstrates the challenges associated with intended lifelong suppressive antibiotic therapy in a highly comorbid patient cohort, including high mortality rates, therapy modifications, and the utility of deprescribing.

One of the key observations of this 10-year follow-up study is a high overall mortality rate of 41% (11/27) since recruitment. Of these deaths, eight occurred within the first five years and a further three during the subsequent five-year period. All deaths were attributed to comorbidities or infections unrelated to their suppressed infection. When assessing the literature, we found no studies with similarly prolonged follow-up encompassing multiple indications. However, previous studies focusing on single indications for suppressive antibiotic therapy have shown a lower rate of all-cause mortality. A mortality rate of 17.4% over 33 months was reported in a cohort of 23 patients with infected prosthetic hip joints [12]. Similarly, another study found a 23% mortality rate among 22 patients with infective endocarditis, with a median follow-up of 8 months [13].

When assessing the comorbidities in our patients over the 10-year period, it was noted that the Charlson Comorbidity Index (CCI) increased across all patients; this is consistent with age-related frailty and the progression of chronic disease. This observation reflects a highly comorbid population at risk for mortality, independent of their antibiotic therapy, suggesting that the observed mortality cannot reasonably be attributed to prolonged antibiotic therapy without further investigation.

The primary inclusion criterion for participant recruitment was an intended course of lifelong suppressive antibiotic therapy. However, the study revealed that in practice this objective was not consistently maintained, with only 56% of participants remaining on therapy at five years and just 26% (7/27) confirmed to be receiving treatment at 10 years. Of the seven patients who were confirmed to remain on antibiotics at 10 years, only four continued their original regimen, while others required modifications due to supply shortages or evolving treatment needs. Notably, no changes to antibiotic therapy were made due to intolerance or adverse effects. A total of six patients were confirmed to have ceased therapy over the study period; this was due to adverse drug reactions (ADRs), projected medication interactions, and stable disease deemed not to require further antibiotic therapy. In all cases, the decision to stop therapy was made collaboratively between the patient and their treating specialist. For five patients, cessation was guided by their infectious disease specialist, while one patient’s therapy was discontinued under the supervision of a non-infectious disease specialist. Notably, none of the patients who ceased therapy had a relapse of their infection or required reinitiation of antibiotics. This result differs from other studies, which show at least some cases of relapsed disease—albeit such studies focus on single-indication therapy with early-stage recruitment [6]. Overall, our data, showing that only 26% of patients remained on their initial regimen, suggests that de-prescribing could be considered as a treatment strategy for patients on suppressive therapy. However, the small sample size limits the ability to identify meaningful predictors for successful de-prescribing, such as infection site, organism, comorbidity burden, or initial regimen. This finding also raises the possibility that some patients may not have required long-term antibiotics for their original infective indications. Similarly, a recent study published in *Open Forum Infectious Diseases* evaluating debridement, suppressive antibiotics, and implantation retention (DAIR) for acute PJI found no significant reduction in treatment failure with suppressive antibiotic therapy (SAT)—concluding that there is no clear benefit in routine SAT [14]. Ultimately, the challenge for clinicians lies in identifying which patients will truly benefit from long-term therapy—and determining both if and when treatment should be discontinued [15]. This study highlights the potential to further explore indications for deprescribing.

A notable decline in long-term follow-up by infectious disease (ID) specialists was observed over the 10-year period. At the time of recruitment, all patients were followed up by the Monash Health infectious diseases department. At five years, only 22% (6/27) of patients remained under active ID supervision; of these, two patients remained under ID follow-up until their deaths, while two others self-discharged but continued antibiotic therapy independently. By 10 years, only 7% (2/27) of patients remained under the care of their infectious disease specialist at Monash Health—both were stable on therapy and reviewed annually. Most patients who remained on antibiotics without ID oversight were managed by their primary care physicians. This indicates a potential shift in management to primary care once patients are stable on suppressive therapy. However, such a transition should only occur after an infectious disease specialist has confirmed the decision to continue long-term therapy with no plan for deprescribing.

This study follows up on a unique cohort of patients, and to our knowledge, no other studies provide the same insight on how long-term suppressive antibiotic therapy for multiple indications, age-related frailty, comorbidities, and time all interrelate. This study, however, is limited by its sample size and reliance on data collected solely from available electronic health records. Three patients did not have any data available on the electronic health records by the 10-year mark; however, they were retained in the original cohort of 27—this ultimately affects internal validity and interpretation of outcomes. Furthermore, the heterogeneity of our cohort, including differing indications and antibiotic regimens, limits our ability to draw specific conclusions. Another important consideration is the absence of routine multidrug-resistant organism screening, which represents a limitation of this study. Given the growing relevance of antimicrobial resistance, future studies should incorporate regular resistance surveillance to better define the long-term risks of suppressive antibiotic therapy. It is also important to note that our data was taken from a single health network and represents long-term antibiotic dispensing records at a large tertiary hospital network and may not reflect prescribing practices in the community, as outlined in other studies [16]. Despite such limitations, this study reinforces that lifelong antibiotic therapy carries risks, including mortality, progressive comorbidities, and treatment burden. However, the finding that patients who ceased antibiotics did not experience relapse suggests that deprescribing should be considered in select patients. A more individualized approach to long-term antibiotic therapy is warranted.

## 4. Methods

As an extension to the original sub-study, this study reports 10-year follow-up of the same cohort identified in 2015. The study was designed as a longitudinal, observational cohort study conducted at Monash Health, a large tertiary-level, university affiliated health service in Melbourne, Australia comprising seven hospital campuses with a catchment that covers 1.8 million residents. Patients were followed for 10 years between April 2015 and March 2025.

The study identified patients, through the hospital drug management system (“Merlin Ver. 4.94”) who were prescribed antibiotics for longer than 12 months [17]. Of these patients, those on long-term antibiotic therapy for infections deemed incurable were enrolled into an observational cohort sub-study, which collected clinical data, conducted a quantitative survey of patient experiences and attitudes towards their therapy. Patients were recruited opportunistically at Infectious Disease outpatient clinics at Monash Medical Centre between April and December 2015 [12]. Exclusion criterion included patients under 18 years of age, those unable to provide informed consent, and patients receiving antibiotic prophylaxis due to immunocompromise or whose antibiotic therapy was dispensed outside the Monash Health network.

Data collection involved a review of medical records to gather baseline demographic characteristics as outcomes including rates of ongoing antibiotic therapy or cessation, adverse events, and mortality. It also recorded isolation of MDROs from clinical specimens, infection control screening swabs and previous swabs conducted in the original sub-study. and isolation of MDROs from clinical specimens, infection control screening and previous study swabs. A Charlson Comorbidity Index (CCI) was calculated as a validated method to identify comorbidities in the population at baseline and the end of the follow-up period. A descriptive analysis was subsequently preformed.

This study was approved by the Monash Health Human Research Ethics Committee (HREC Ref: 14379A)

## 5. Conclusions

This 10-year follow-up study of a cohort of patients on lifelong antibiotics provides insights into the long-term outcomes of this practice and highlights the challenges and complexities of this therapy. While some patients may benefit from sustained infection suppression, the increasing comorbidities and low relapse rates after therapy cessation suggest that prolonged antibiotic therapy may not always be necessary. Importantly, this study highlights successful deprescribing in select patients without infection relapse, underscoring the need to re-evaluate long-term antibiotic use on an individualized basis. Future research should focus more on formulating deprescribing protocols to optimize patient outcomes while minimizing long-term risks.

## Figures and Tables

**Table 1:** Patient characteristics of the original cohort (time of recruitment to 10-year follow-up).

Age (Baseline)	Sex	Indication	Targeted Organism	Antibiotic Regiment	Treatment Adjustment/Reason—5 Years	Treatment Adjustment/Reason—10 Years	CCI Baseline	CCI—5-Year Follow-Up	CCI—10-Year Follow-Up	MDRO—Isolate Site	Cause of Death	Hospital Admission Days ( Total/Infection-Related/ICU) Year 5–10	Ongoing ID Follow-Up—10-Years	ADRs
78	M	PJI	MRSA	Rifampicin, Fusidic Acid	No change	Ongoing Abx—till time of death	5	13	13	MRSA, ESBL K. pneumoniae—Knee tissue	Aspiration pneumonia	28/14/0	Yes	
71	M	Post-laminectomy infection	*E. cloacae*, *P. mirabilis*	Ciprofloxacin	No change	Unclear if ongoing at time of death	7	7	No data available	N/A	Deceased (unknown cause)		No	
69	M	PJI	MRSA	Rifampicin, Fusidic Acid	No change	Ongoing Abx—till time of death	2	3	10	MRSA—joint tissue	Metastatic Caecal cancer	53/0/0	Yes—once yearly reviews	Stable
42	F	Recurrent Urinary tract infection	*E. faecalis*	Nitrofurantoin	No change	Ceased by a non-ID specialist	1	1	2	N/A	N/A	0/0/0	No	
74	M	VGI	*S. typhimurium*, VRE	Amoxicillin/Clavulanic acid, Pristinamycin	Changed amoxicillin/clavulanic acid to amoxicillin/ADR	Ceased Pristinamycin during shortage, amoxicillin monotherapy	6	10	10	VRE, CRE, E. cloacae—screening swab	N/A	6/0/0/admitted to different hospitals	Yes	Diarrhea
77	M	PJI	MSSA	Cephalexin	No change	Ongoing Abx	4	5	6	N/A	N/A	16/0/0	No	
45	F	CIEDI	*C. acnes*	Penicillin	No change	Ongoing Abx	1	3	3	N/A	N/A	1/0/0	No	
69	F	PJI	MRSA	Rifampicin, Fusidic Acid	No change	Ongoing Abx	3	4	6	MRSA—joint tissue	N/A	0/0/0	Self-discharged from clinic—cancer diagnosis	Stable
59	F	PJI	*S. agalactiae*	Cephalexin	No change	Ongoing Abx	6	6	8	N/A	N/A	82/0/0	No	Vaginal thrush
45	F	PJI	*S. epidermidis*, MSSA	Rifampicin, Fusidic Acid	No change	Ongoing Abx	1	2	3	N/A	N/A	2/2/00	Yes—6 monthly reviews	N/A
65	F	PJI	MSSA, *C. aurinmucosum*	Flucloxacillin, Ciprofloxacin	Changed to doxycycline/Failed definitive surgery, new target organism	Ongoing Abx	5	5	6	VRE—screening swab	N/A	38/0/0	No—FTA from clinic (2023)	
56	F	PJI	MSSA	Cephalexin	Ceased—no longer required	Remains off antibiotics	4	5	6	N/A	N/A	2/0/0	No	Vaginal thrush
65	M	Recurrent MSSA bacteraemia	MSSA	Cephalexin	Ceased—no longer necessary	Remains off antibiotics	3	7	7	VRE—screening swab	N/A	9/5/00	No	N/A
59	M	VGI	*S. epidermidis*	Clindamycin, Amoxycillin	Ceased—unclear reason	Remains off antibiotics	3	4	4	N/A	N/A	3/3/00	No	
68	M	VGI	*S. maltophilia*, VRE, *P. moteilli*, *C. Albicans*	Pristinamycin, Co-trimoxazole, Ciprofloxacin, Fluconazole	Ceased—ADR	Remains off antibiotics	4	7	8	VRE—abdominal pus ESBL K. pneumoniae—screening swab	N/A	9/0/0	No	Acute kidney and liver injury
65	M	PJI	MRSA	Rifampicin, Fusidic Acid	Ceased—projected medication interactions	Remains off antibiotics	5	5	7	MRSA—joint tissue	N/A	0/0/0	No	Diarrhea
88	F	Recurrent MSSA bacteraemia, OM	MSSA	Cephalexin	No change	Unclear if ongoing	7	7	No data available	N/A	N/A	No data available	No	
86	F	PJI	MRSA	Rifampicin, Fusidic Acid	No change	Unclear if ongoing	9	9	No data available	N/A	N/A	No data available	No	
73	F	Infected spinal metalware	PSSA	Amoxycillin	No change	Unclear if ongoing	9	9	No data available	N/A	N/A	No data available	No	

M—male; F—female; PJI—prosthetic joint infection; PVI—prosthetic valve infection; MSSA—methicillin-susceptible Staphylococcus aureus; PSSA—penicillin-susceptible Staphylococcus aureus; OM—osteomyelitis; VGI—vascular graft infection; MDRO—multi-drug resistant microorganisms; MRSA—methicillin-resistant Staphylococcus aureus; VRE—vancomycin-resistant Enterococci; CRE—carbapenem-resistant Enterobacteriaceae; ICU—intensive care unit; ADR—adverse drug reaction. Charlson Comorbidity Index—baseline refers to the time of recruitment to the study.

## Data Availability

The data used in this study have been presented in table format in the results section. Any further deidentified information can be made available in deidentified format upon reasonable request.
